# Diagnosis of cross-reactivity in patients with birth pollinosis

**DOI:** 10.1186/2045-7022-1-S1-P67

**Published:** 2011-08-12

**Authors:** Katarzyna Napiórkowska, Zbigniew Bartuzi, Magdalena Zbikowska-Gotz, Malgorzata Graczyk, Ewa Szynkiewicz

**Affiliations:** 1 Collegium Medicum in Bydgoszcz, Department of Allergology, Clinical Immunology and Internal Diseases, Bydgoszcz, Poland

## Background

Patients with the birch pollen allergy frequently develop hypersensitive reactions to certain plant food. Mostly, it is caused by cross- reactivity. Diagnosis of this allergy became possible due to such method as immunoblotting.

## Objective

The aim of this study was to investigate the role of cross-reactivity and the diagnostic value of skin test (using commercial and native extracts of allergens), total and specific IgE and immunoblotting method for patients with pollinosis.

## Material and Methods

The clinical history and the positive SPT with the birch extract were the condition for qualifications. 23 patients included in the first group were only birch allergic. 35 patients in the other group had birch pollen allergy and they reported symptoms after eating apple, celery, carrot, tomato, banana, peach, peanut and hazelnut. The skin tests were performed and serum IgE concentration (total and specific) were determined for all individuals. The immunoblotting was performed for the patients with the positive value of birch, apple, celery and/or carrot specific IgE.

## Results

Patients sensitive to birch pollen,with coexisting food allergy, had most often symptoms after eating apples, hazelnuts and of peaches, and less frequently after carrots, celery, peanuts, tomatoes and bananas. Sera of 12 patients revealed the reaction against the birch pollen protein with a molecular weight 17-18 kDa corresponding to Bet v 1. Sera of 2 patients revealed the presence of antibodies cross-reacting with the apple protein which may indicate Mal d 1. Serum of 6 patient revealed the presence of antibodies cross-reacting with apple and celery protein, which may indicate Mal d 1 and Api g 1. Serum of only one patient revealed the presence of antibodies cross-reacting with the apple, celery and carrot protein, which may correspond Mal d 1, Api g 1 and Dau c 1. Sera of 6 persons demonstrated the presence of antibodies reacting with apple protein with the molecular weight 10kDa which may correspond the LTP.

## Conclusions

Although the immunoblotting is an effective method confirming cross-reactivity, it still remains the method of verifying and supplementing other tests.

